# *In Vitro* Evaluation of Fluorescence Glucose Biosensor Response

**DOI:** 10.3390/s140712127

**Published:** 2014-07-08

**Authors:** Mamdouh Aloraefy, T. Joshua Pfefer, Jessica C. Ramella-Roman, Kim E. Sapsford

**Affiliations:** 1 Center for Devices and Radiological Health, Food and Drug Administration, Silver Spring, MD 20993, USA; E-Mails: Mamdouh.Aloraefy@fda.hhs.gov (M.A.); Kim.Sapsford@fda.hhs.gov (K.S.); 2 Department of Biomedical Engineering, Catholic University of America, Washington, DC 20064, USA; 3 Department of Biomedical Engineering and Herbert Wertheim College of Medicine, Florida International University, Miami, FL 33174, USA; E-Mail: jramella@fiu.edu

**Keywords:** affinity biosensor, competitive binding, concanavalin A, continuous glucose monitoring, fluorescence sensor, FRET-based, glucose sensor, minimally-invasive, optical

## Abstract

Rapid, accurate, and minimally-invasive glucose biosensors based on Förster Resonance Energy Transfer (FRET) for glucose measurement have the potential to enhance diabetes control. However, a standard set of *in vitro* approaches for evaluating optical glucose biosensor response under controlled conditions would facilitate technological innovation and clinical translation. Towards this end, we have identified key characteristics and response test methods, fabricated FRET-based glucose biosensors, and characterized biosensor performance using these test methods. The biosensors were based on competitive binding between dextran and glucose to concanavalin A and incorporated long-wavelength fluorescence dye pairs. Testing characteristics included spectral response, linearity, sensitivity, limit of detection, kinetic response, reversibility, stability, precision, and accuracy. The biosensor demonstrated a fluorescence change of 45% in the presence of 400 mg/dL glucose, a mean absolute relative difference of less than 11%, a limit of detection of 25 mg/dL, a response time of 15 min, and a decay in fluorescence intensity of 72% over 30 days. The battery of tests presented here for objective, quantitative *in vitro* evaluation of FRET glucose biosensors performance have the potential to form the basis of future consensus standards. By implementing these test methods for a long-visible-wavelength biosensor, we were able to demonstrate strengths and weaknesses with a new level of thoroughness and rigor.

## 1. Introduction

Diabetes mellitus is a growing epidemic with a prevalence rate of 346 million worldwide. In 2004, an estimated 3.4 million people died from the consequences of high blood sugar, and it has been projected that diabetes deaths will double between 2005 and 2030 [1]. In the United States, 25.8 million adults and children live with diabetes [2]. Diabetes control involves glucose management, including self-monitoring and maintenance of glucose levels within normal ranges (preprandial: 70–130 mg/dL; postprandial: <180 mg/dL; higher concentrations represent hyperglycemia) [3]. Yet glucose test systems currently used for self-monitoring cause skin irritations, are invasive, and/or require frequent calibration using glucometers associated with skin-pricks that reduces patient compliance [4].

Although rapid and accurate monitoring of blood glucose levels is crucial for many patients with diabetes as well as non-diabetics in critical care settings, current modalities are invasive or lack continuity. A variety of substitutes that enable non-invasive measurements have been researched for continuous glucose sensing, among which are optical approaches such as near- and mid-infrared absorption, Raman, Fourier transform infrared, photoacoustic, and ocular spectroscopy, and optical coherence tomography [5–10]. However, few technologies have been verified to be suitable for *in vivo* application due to a variety of challenges. These include lack of endogenous signal, excessive interference from non-glucose constituents (absorbers and scatterers), lack of sensitivity to physiologically-relevant glucose concentrations (GCs), or movement artifacts [11–17]. One promising technique for *in vivo* glucose measurement is a biosensor that—once implanted—can provide real-time, non-invasive measurements of GC via optical interrogation approaches. While a variety of techniques have been explored for this class of biosensors, including time-resolved fluorescence of sol-gel immobilized glucose oxidase [18] and Förster (or fluorescence) resonance energy transfer (FRET), the latter approach has shown the greatest potential to lead to innovative devices for improved patient care during home and hospital use [5,19,20].

FRET glucose biosensors often involve competition between glucose and a carbohydrate derivative for binding sites [21,22]. A well-established format is based on a natural glucose-binding protein (lectin) concanavalin A (ConA) that is fluorescently labeled [14]. In the absence of glucose, the donor-labeled dextran molecules are bound to the sites of the acceptor-labeled ConA, bringing both fluorophores in proximity enough for FRET-based quenching to occur. As glucose, to which the binding sites have a higher affinity, increases, it displaces dextran molecules. Thus, the signal from the liberated dextran-attached label is recovered due to reduced FRET, providing an indirect quantification of GC [23]. The system is reversible and the lectin-ligand binding produces a glucose-dependent modulation in energy transfer between donor and acceptor dyes, allowing a continuous transduction of a detectable signal by the fluorescently-labeled system.

The biosensor may reside under the skin, in dermal tissue [24], or in the eye [25], to interact with the interstitial or aqueous humor glucose, and continuously monitor glucose levels, which are correlated to plasma glucose [26–28]. In an attempt to remedy the losses of fluorescence signal due to tissue absorption and scattering, long-wavelength dyes have been used [20,29,30]. The use of hydrogel-based polymers has been reported for the immobilization of receptor molecules and suggested to improve diffusion of small molecules, such as glucose, and enhance signal-to-noise ratio (SNR), biocombatibility, and stability of implanted glucose sensors [19,31]. Permeability-controlled hydrogel pads using Layer-by-Layer (LBL) self-assembly have been developed to enhance encapsulation efficiency and selectivity [32].

Although a wide variety of test methods for evaluating optical glucose biosensor performance have been implemented in prior studies, there is little consistency between studies and no consensus has been achieved on an optimal battery of approaches. Some of the performance characteristics that have been quantified in individual glucose biosensor studies include: spectral response, calibration curve, kinetic response and short-term stability (48 h) [19]; long-term stability (110 days) [30]; kinetic response [32]; spectral response, calibration curve, kinetic response, and short-term stability (37 h) [33]; and mean absolute relative difference (MARD) and Clarke's error grid analysis [34]. Progress in development and translation of novel optical biosensors would likely benefit from the publication of consensus documents that describe standardized performance test methods, such as those which have been developed previously for glucose monitoring systems (especially electrochemical) [35,36]. Therefore, the purpose of this research was to investigate *in vitro* test methods for FRET biosensor performance evaluation that may form the basis of future standards. While the introduction of a novel biosensor is not the intent of this study, our results do provide a more thorough *in vitro* characterization of biosensor performance than has been provided for similar devices in the literature. Specifically, the aims of this study were: (1) to identify a set of performance test methods based on optical biosensor literature and techniques adapted from relevant standards; (2) to fabricate an effective FRET biosensor; and (3) to implement the aforementioned test methods for quantitative characterization of biosensor response.

While *in vivo* testing is critical for establishing device effectiveness and *in vitro* environments do not represent as strong a challenge, the availability of a set of well-validated protocols for preclinical testing that aligns with recognized consensus standards can provide useful insights into performance. This will facilitate evaluation of optical glucose biosensors throughout the development process and promote technological innovation, thus hastening realization of new clinical options for diabetics.

## 2. Experimental Section

### 2.1. Fabrication of FRET Glucose Biosensor

Our protocol for the fabrication of a FRET glucose biosensor based on long visible wavelength fluorescence is described below. Alexa Fluor 594 (AF594, 1 mg, absorption/emission maxima ∼590/617 nm, Life Technologies, Grand Island, NY, USA) and Alexa Fluor 647 (AF647, absorption/emission maxima ∼650/668 nm, Invitrogen, Grand Island, NY, USA) were incorporated as the dextran-labeling donor and ConA-labeling acceptor, respectively. Since a dextran molecule with a sufficiently low molecular weight to retain within the hydrogel was needed [37], we used the 70 kDa amino-dextran (Life Technologies). The dextran was dissolved in a dimethyl sulfoxide (DMSO, 100 mL, Sigma Aldrich, St. Louis, MO, USA) and labeled with the donor dye AF549 using two spin desalting columns and a washing solution of phosphate buffered saline (PBS, 1 mL) to produce solution of AF594-dextran 70 kDa (41.4 μM). The dependence of fluorescence intensity on the concentration of the donor fluorophore conjugate was quantified. To ensure the measurements are conducted in a fluorescence interval that varies linearly with donor concentration, dilutions of AF594-dextran were generated using a base concentration of 2.76 μM.

A protein conjugate stock of 45 μM was prepared by dissolving the AF647-ConA (5-mg vial, Life Technologies) in 1 ml of 0.1 M Tris buffer and further diluted to prepare the working concentration, using buffer modified with 0.1% (*w*/*v* percent) calcium chloride (CaCl_2_, Sigma Aldrich) and 0.1% (*w*/*v* percent) manganese chloride (MnCl_2_, Sigma). The mixture was centrifuged briefly before each use and only the clear portion extracted for sample preparation to eliminate any protein aggregates that may have formed during storage, thus reducing nonspecific background staining.

To immobilize receptor proteins and create a molecular recognition interface to enhance glucose selectivity and response [38], we formed the pair complex in a hydrogel-based system. The hydrogel used in this work was a low-melting temperature agarose (100 g, Fisher Scientific, Pittsburgh, PA, USA), a porous polymer that can be dissolved in warm buffer but gel on cooling (<35 °C). Various hydrogel concentrations were investigated to observe the influence of its volume on the glucose response.

### 2.2. Layer-by-Layer (LBL) Self-Assembly Process

We additionally coated our hydrogel surfaces, using a polyelectrolyte LBL self-assembly process to further retain biosensor sensing constituents and enhance glucose selectivity through a permeability-controlled membrane. This approach involved coating the hydrogel with alternating charged films of poly(allylamine hydrochloride) (PAH, Sigma Aldrich) and poly(sodium 4-styrene-sulfonate) (PSS, Sigma Aldrich). The PAH was dissolved in 0.1 M Tris buffer at a concentration of 0.25 g per 50 mL and added at volume of 100 μL. The components were incubated at room temperature for 15 min and then washed twice, using 100 μL Tris buffer. Using the same protocol, PSS was added (100 μL of 5 mg/mL); samples were incubated then washed. This formed a polymeric microcapsule of one-layer film and the process was repeated for additional layers.

### 2.3. Trade-off Analysis of Biosensor Components

Key parameters of the biosensor were experimentally evaluated in trade-off analysis. The acceptor-to-donor (A/D) ratio was analyzed to determine the optimal glucose sensitivity. The complex mixture was dissolved in the modified buffer to make solutions with final volumes of 150 μL. Polymeric hydrogel-based biosensors incorporating complex pair at A/D molar ratios of 1:1, 2.5:1, 5:1, 10:1, and 20:1 were fabricated and tested (with and without glucose) for optimum glucose response.

Since sensing components are prone to loss by diffusion during the LBL self-assembly process, we evaluated the encapsulation efficiency and effect of washing using four sets of biosensors, with 0- to 4-layers LBL. The response was observed by measuring the fluorescence after the addition of each layer.

### 2.4. Fluorescence Measurements and FRET Quantification

Fluorescence intensity of the biosensor was measured with a fluorescence microplate reader (Infinite M1000, TECAN, Morrisville, NC, USA) as follows. Upon completion of the LBL process, a 100 μL buffer solution was added to each well, and fluorescence intensities were recorded with and without glucose. Glucose solutions were prepared in Tris buffer over a range of concentrations (0–600 mg/dL) and loaded into designated hydrogel-containing wells at 100 μL volumes. For all fluorescence measurements performed to calculate GCs, excitation and emission wavelengths of 590 and 617 nm, respectively, were used. Both excitation and emission bandwidths were 5 nm.

### 2.5. Test Methods and Response Characteristics

In this section, we describe the proposed response test methods in terms of their general performance characteristic, specific experimental/analytical methodologies, and the metrics used to quantify performance. The identification of best practices was complicated by a lack of consistency in characteristics and terminology recommended in different references. The consensus documents reviewed here are focused primarily on *in vivo* testing; however, we have adapted several test methods for *in vitro* testing, where appropriate. Two of the key standards used were the Clinical and Laboratory Standards Institute (CLSI) POCT5-A: Approved Guideline, and the International Organization for Standardization (ISO) 15197:2003 [35,36]. The CLSI document recommends evaluation of accuracy, sensitivity and specificity, device stability, calibration, lag time, and trueness of measurement and device traceability. The key testing characteristics recommended by the ISO standard are repeatability, intermediate precision, and analytical accuracy.

The full list (Table 1) of the *in vitro* response characteristics implemented in this study includes: spectral response, linearity, analytical sensitivity, detection capability, kinetic response and lag time, reversibility, stability, precision, accuracy, error analysis, and comparative accuracy. It should be noted that this Table lacks a key performance characteristic, interference. The potential for GC values to be altered by non-glucose sugars and medications is beyond the scope of this study, due to its extensive methods and results, and will be addressed in a future publication currently under preparation. Furthermore, while performance threshold values are not listed in this table—and may require additional research since the correlation between *in vitro* and *in vivo* performance is not well established—potential criteria are discussed in Section 3.

#### 2.5.1. Spectral Response

The spectral fluorescence distributions provide a more thorough illustration of FRET biosensor output than the narrow bandwidth measurements that were used for GC estimation. We evaluated spectral response over a range of GCs from 0 to 400 mg/dL. The fluorescence reader described in Section 2.4 was used to generate spectra based on an excitation wavelength of 590 nm and detection range of 610–750 nm. This covers most of the range of the donor fluorescence (although some was cut off by the emission filter), including the range used for calibration and the full range of acceptor fluorescence.

#### 2.5.2. Calibration Quality

To construct a calibration curve, we prepared a control solution and eleven positive known samples. The standard GCs included 40–320 mg/dL (in an increment of 40) and 400 mg/dL, 500 mg/dL, and 600 mg/dL. A dose-response relation was established by measuring the biosensor fluorescence intensity for the entire glucose range. Total of 48 biosensors were fabricated and a control solution as well as eleven standard solutions of known GCs prepared. Four replicate biosensors were used to measure each concentration. The results were used to construct a calibration curve and the linearity of the regression curve and its fit quality were evaluated in terms of *r*^2^ value [19]. The analytical sensitivity was calculated from the slope of the calibration curve as a percentage change in fluorescence intensity per GC.

#### 2.5.3. Limit of Detection

Our protocol for characterizing the biosensor's detection capability focuses on the lowest concentration of glucose that can be detected, which defines the limit of detection (LoD) expressed as the glucose concentration (*x*-axis), and is derived from the smallest fluorescence (*y*-axis, *F_L_*) that can be detected with reasonable certainty [51]. This test was based on CLSI EP17-A2 [40] and involved 60 total control samples (four glucose-free samples with five replicates across three days) and 60 total positive replicates (four increasingly lower glucose content samples with five replicates over three days). As the LoD is an estimate of the lowest level of the calibration curve, and since concentrations that are 1–5 times the estimated detection level are conventionally acceptable values of low-level glucose to examine, our spiking low-content GCs included 10 mg/dL, 15 mg/dL, 20 mg/dL, and 25 mg/dL. A sample mean with *p* < 0.05 was considered as significantly different (α = 0.05).

#### 2.5.4. Kinetic Response

The time required for changes in GC to be detected and reported by a biosensor can significantly impact patient health. A key metric, lag time, is often described in terms of the duration required for the sensor's glucose measurement to become equal to that of the reference value [35,52]. In our testing, we defined response time as the duration required for the biosensor-predicted GC value to traverse 90% of the difference between initial and final reference GC values. We monitored the variation in fluorescence with time upon a step change in GC from 0 to 50, 100, 200, or 400 mg/dL, or from these values to 0 mg/dL upon incubation in buffer to regenerate the FRET-based quenching.

#### 2.5.5. Reversibility

The ability of the biosensor to accurately respond to increases and decreases in GC with minimal hysteresis demonstrates its reversibility. This was characterized by recording the steady state response while elevating GC up to 400 mg/dL in 100 mg/dL steps. Subsequently, GC was decreased in a similar manner. To quantify any irregularities in fluorescence response following reverses in GC, signal responses was measured and graphed.

#### 2.5.6. Stability

The lack of variation in fluorescence intensity over time defines the sensor stability [35,53], which can be affected by a variety of factors including the loss or aggregation of sensing components. To evaluate long-term changes in maximum fluorescence intensity, two sets of biosensors were tested for stability. One set was placed in washing buffer and the other was periodically measured at a GC of 50 mg/dL. Testing was performed for each biosensor set over a period of 30 days. Biosensors in both sets were stored in washing buffer at 4 °C. The percentage deviation was reported to demonstrate the signal fluctuations of the biosensor over the stable range.

#### 2.5.7. Precision

Precision measures the level of agreement among independent test results obtained under constant specified conditions [36]. Repeatability is described as within-run precision of results, obtained with the same method on identical test items in the same laboratory by the same operator using the same equipment within short intervals of time [54]. The common statistical indicator is the coefficient of variation (%CV) calculated as 100 × SD/mean, where SD is the repeatability (within-run) standard deviation. Our evaluation of repeatability involved a fabrication of three 20-biosensor sets and was based on the ISO 15197 criteria. Each set was exposed to different (50, 80 and 120 mg/dL) GCs and the response recorded. The mean ± SD for each level was analyzed and the CV calculated.

#### 2.5.8. Bias

The level of agreement between the measured GC and its true quantity characterizes the accuracy of a test method [35]. The bias—systematic measurement deviation when a large series of test results is considered [36]—is determined through direct comparison of the glucose measurements with reference values (or, known concentrations, for aqueous solution glucose). Difference plots are the recommended approach for depicting system accuracy because statistical assumptions are minimal and the percentage of data points meeting the system accuracy criteria, as well as estimating bias, are easily calculated [36]. The fluorescence intensity produced by the biosensor when exposed to known samples were measured and the calibration equation used to predict the corresponding GCs (*n* = 29). We quantified the difference between individual test results of biosensor measurement and reference values. The difference (*y*-axis) *vs.* true GCs (*x*-axis) was plotted and the total error reported. The ISO 15197 percentage of results for GCs ≤ 75 mg/dL and that for higher glucose levels were also reported.

The average error, however, tends to overlook outliers, in spite of their potential to affect treatment decisions [55]. To identify extreme values, a boxplot chart [46] (also called Box and Whiskers plot, which includes median, range, and outliers) was used. It is an improved version of the frequency distribution, as it is based on median deviation, and thus is less sensitive to outliers than a mean-based approach. This approach provides descriptive statistics and visual comparison among distributions of data in a box shape diagram [49].

#### 2.5.9. Error Analysis

While analytical accuracy represents the statistical deviation from reference measurements, it is critical to judge the measurement performance against a clinically acceptable criterion. Clarke's error grid analysis (EGA) has been a common approach for evaluating the severity of a deviation-related error in measurements and its potential to alter the treatment decision [49]. The grid is a graph of reference against the test method data pairs, which is divided into five zones of varying degrees of inaccuracy of glucose estimations, defined on scatter plot, with the *x*-axis as the reference glucose and *y*-axis as values generated by investigated system [50]. The results of validation data generated by the biosensor (*n* = 29) was subjected to EGA and the clinical significance of differences between its measurements and actual GC (through 400 mg/dL) assessed.

#### 2.5.10. Comparative Accuracy

This evaluation of accuracy involves comparison of the biosensor to a commercially available hand-held electrochemical glucose meter. To assess how our biosensor's response differs from the established methodology, the Bland-Altman difference plot—a classical approach for analyzing the corresponding results from different methods [56]—was used. Measurements from the biosensor were compared with those obtained by the commercial meter. Seven samples containing GCs up to 400 mg/dL were tested, each of which was measured four times. The samples (*n* = 28) were first measured with both methods, and the difference (bias) between both measurements (*y*-axis) as a function of the mean of the two readings of each sample (*x*-axis) was then plotted. Additional reference lines—zero bias and 95% upper (0 + 1.96 SD difference) and 95% lower (0 − 1.96 SD difference) limits—were also overlaid on the plot.

## 3. Results and Discussion

### 3.1. Biosensor Design: Trade-off Analysis of Critical Characteristics

Key characteristics of biosensor elements were experimentally evaluated, and the results underwent a trade-off analysis to determine the optimal design. The evaluation included dextran molecular weight, hydrogel concentration, A/D ratio, and number of polymeric layers. A 70 kDa-dextran was identified as the optimal molecular weight—low enough to minimize large branches, yet high enough to retain materials within the hydrogel. A hydrogel concentration of 2% was chosen, as higher concentration produced no significant increase in signal intensity, but higher concentrations may reduce dextran-lectin dissociation and association. The result from evaluating the effect of A/D ratio on total fluorescence signal showed that a 10:1 ratio of ConA to dextran provided moderately high signal and 45% variation in signal intensity with the addition of glucose. Furthermore, the use of a higher ratio may cause aggregation.

The evaluation of the effect of frequent washing over the course of the two-hours LBL self-assembly process included biosensors with one, two, three, or four full polymeric layers. Sensing components losses were reflected in signal decay, which was 73% for no layers, 51 for one layer, 30% for two layers, 28% for three layers, and 27% for four layers. Therefore, biosensors with two polyelectrolyte layers were used in subsequent measurements as additional layers would provide negligible benefit but may reduce the glucose diffusion rate.

### 3.2. Biosensor Response Characteristics

The biosensor response has been evaluated and analysis of performance data is presented and comparisons made to available criteria.

#### 3.2.1. Fluorescence Output

**Figure 1.**
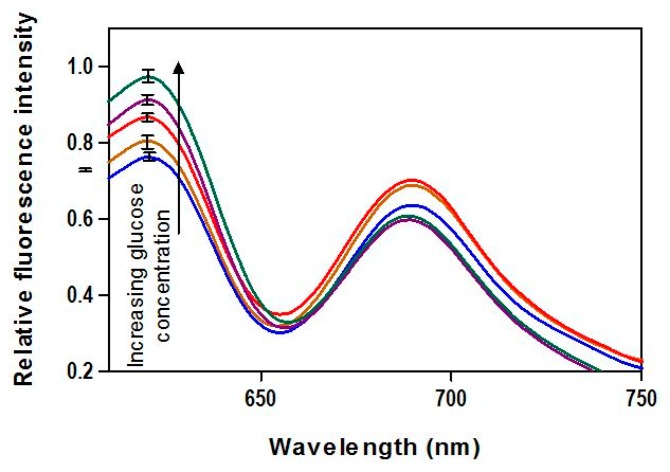
Fluorescence emission spectra of AF594 conjugate incorporated in FRET-based biosensor, in the presence of GCs of 0, 100, 200, 300, and 400 mg/dL. Error bars at the emission peak are shown (*n* = 4).

The emission spectra (Figure 1) upon exposure to various glucose concentrations demonstrated the viability of AF594/AF647 pair complex incorporated in FRET-based biosensor for creating a FRET-based biosensor capable of detecting glucose. The donor fluorescence showed a peak emission at 620 nm and increased monotonically with increasing GC. No significant irregularities in the spectra, such as spectral shifts due to the inner filter effect (fluorescence absorption and re-emission), are apparent.

#### 3.2.2. Calibration Quality

**Figure 2.**
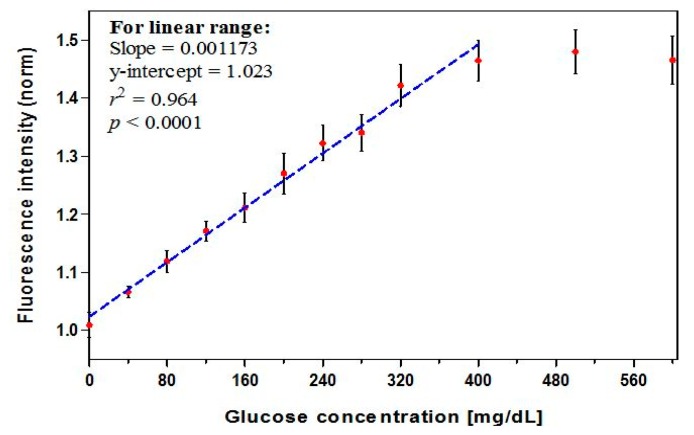
Calibration data illustrate the biosensor's responses to GCs between 0 and 600 mg/dL. The dotted regression line demonstrated a linear range, up to GC of 400 mg/dL.

The dose-response curve (Figure 2, *n* = 12) showed a linear relationship through 400 mg/dL (*r^2^* = 0.964). A maximum intensity change of 45% was found to occur in the presence of 400 mg/dL glucose. A high correlation coefficient (*r*) of 0.99 or larger is often used as an acceptance criterion for linearity. However, this is not sufficient to prove that a linear relationship exists, and a method with *r* of slightly less than 0.99 can be viable [57]. The linear range covers the clinically-relevant concentrations (preprandial: 70–130 mg/dL for glycemic control and >130 mg/dL for hyperglycemia; postprandial: <180 mg/dL for glycemic control and >180 mg/dL for hyperglycemia) [3]. A leveling off, or saturation, of the signal was observed as GC exceeded 400 mg/dL, an effect that has been seen in prior studies and is likely due to a high level of occupied protein binding sites [39,52,53,58]. Thus 400 mg/dL represents the limit of the useful range of the biosensor, and accuracy may degrade slightly for GCs at the very upper end of this range. The calibration equation for the linear range, expressed as the glucose concentration (mg/dL), was derived from the regression equation and calculated as:

(1) GC=(F−1.023)/0.001173

where *F* is the normalized fluorescent intensity measured at emission wavelength of 617 nm. This equation was used for the prediction of glucose, expressed as concentration in units of mg/dL.

The analytical sensitivity—a characteristic parameter indicating how much change in the biosensor detected signal per unit change in GC [35]—was estimated to be 0.12% per mg/dL over the linear range of 0–400 mg/dL glucose. The current sensitivity exceeds that found in some prior results (0.04% per mg/dL) [58], but is lower than that found in others (0.18% per mg/dL) [53], for similar measurement ranges.

#### 3.2.3. Limit of Detection (LoD)

The mean*_B_* ± SD*_B_* fluorescence of all control (*n* = 60) results in the dataset was 1.025 ± 0.0035, where SD*_B_* is the pooled standard deviation. To obtain the value of limit of detection of our biosensor, we first calculated the limit of control, expressed in fluorescence, as follows:

(2) $${\text{LoB}}_{F}={\text{mean}}_{B}+{\text{c}}_{p}{\text{SD}}_{B}$$

(3) $${\text{c}}_{p}=1.645/(1-(1/(B-K)))$$

where, c*_p_* (=1.67) is a multiplier based on the confidence level chosen.

The value 1.645 corresponds to a confidence level of 95%, *B* (=60) is the total number of control results, and *K* (=4) is the number of control samples. Therefore, the fluorescence limit of control when glucose-free samples were tested is 1.031, which represents the highest fluorescence from control measurements that can be observed with a 95% probability.

The value of the pooled standard deviation (SD*_L_*) across the low level glucose samples was ±0.013. The critical signal limit of low level samples, expressed as the fluorescence measurement, was determined through the following equation:

(4) $$F_{L}={\text{LoB}}_{F}+{\text{c}}_{p}{\text{SD}}_{L}$$

(5) $${\text{c}}_{p}=1.67=1.645/(1-(1/(L-J)))$$

where *J* (=4) is the number of low level samples and *L* (=60) is the total number of all low level sample results. This critical fluorescence limit was calculated to be 1.053, which represents the lowest biosensor output that can be statistically (α = 0.05) discriminated from the background.

The calibration equation (Equation (1)) was used to convert the response variable (fluorescent, dimensionless, *y*-axis) into GC (mg/dL, *x*-axis). Consequently, the limit of detection, defined as the minimum concentration, which corresponds to the smallest fluorescence that was detectably different than 0 at the specified confidence, was approximated as 25 mg/dL. This concentration equivalent represents our biosensor LoD and reflects the minimum glucose level that could be statistically detected, with a 95% confidence interval of 24.71–26.25 mg/dL, for the true positive sample.

#### 3.2.4. Kinetic Response

**Figure 3.**
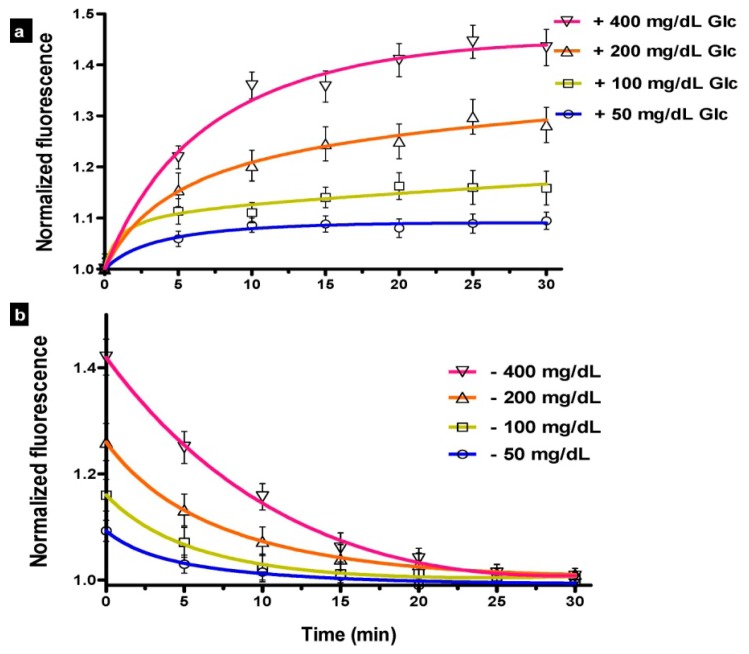
Kinetic study, demonstrating: (**a**) Step response in the presence of 50, 100, 200, and 400 mg/dL glucose; and (**b**) Regeneration of FRET-based quenching in the absence of glucose.

The kinetic response (Figure 3) demonstrated the variation in predicted glucose level over time in response to (a) various step inputs of glucose; and (b) incubation in washing buffer. The fluorescence intensity was normalized to the measured signal before adding the glucose, at T = 0 min. The 90% response was reached in 12 min, 15 min, 16 min, and 18 min with the introduction of 50 mg/dL, 100 mg/dL, 200 mg/dL, and 400 mg/dL of glucose, respectively; whereas the durations for the 90% signal decay with washing buffer were 13 min, 15 min, 18 min, and 19 min, respectively. The measurements were fitted into a curve, which was analyzed to interpolate the 90% values. These values are similar to those measured previously for FRET biosensors [27].

The results reported here reflect the sensor lag, mainly due to diffusion of the glucose into the sensor. Since the current study focuses on *in vitro* performance, the physiological component of lag, which represents the time difference between blood and interstitial glucose [35], was not accounted for in our evaluation. Additionally, while processing delay—which may result from averaging or otherwise processing multiple measurements, can have non-negligible contribution to lag time for FRET biosensors, it was not considered in our measurements. The maximum rate of change in blood glucose levels over time has been estimated to be 2 to 4 mg/dL/min (based on 90% to 99% confidence intervals) [35] and physiologically-induced lag due to the plasma to interstitial fluid glucose transition has been estimated to be approximately 5 min [59–61].

#### 3.2.5. Reversibility

**Figure 4.**
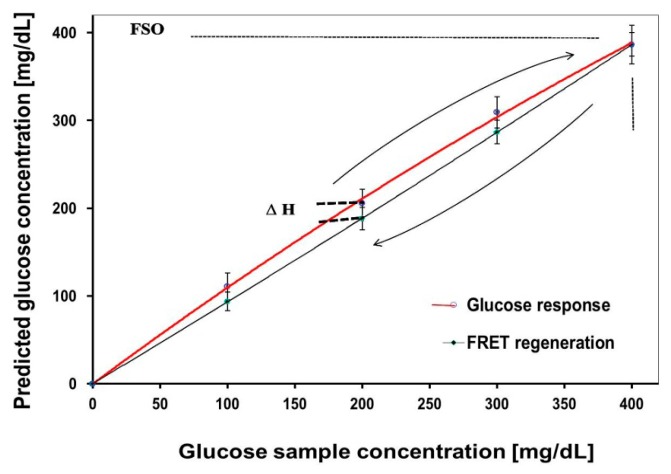
Biosensor reversibility over the course of incremental increases in glucose concentration (100 mg/dL) to a level of 400 mg/dL, and subsequent return to baseline.

The predicted glucose levels against known glucose values are shown in Figure 4, for both increasing and decreasing glucose levels. The former demonstrates a signal increase by unbound-dextran labels in response to incremental (100 mg/dL) elevation of GCs up to 400 mg/dL, and the latter reflects a FRET-based signal quenching due to increasing ConA-dextran complex in the absence of glucose. The biosensor demonstrated almost full reversibility by returning to baseline at the end of the decreasing cycle. This is generally due to the fact that the protein-ligand binding occurs between non-polar regions of both, without an involvement of covalent bonds. The plot showed a hysteresis (ΔH) of ∼17 mg/dL, with maximal hysteresis error (ΔH/FSO) of 0.046 across the entire range of glucose, where FSO (full scale output) represents the maximum linear output. Analyses in prior publications that have addressed reversibility were primarily qualitative [30], but rigorous quantification of results was not provided. In general, the hysteresis should not be greater than the MARD or LOD.

#### 3.2.6. Stability

**Figure 5.**
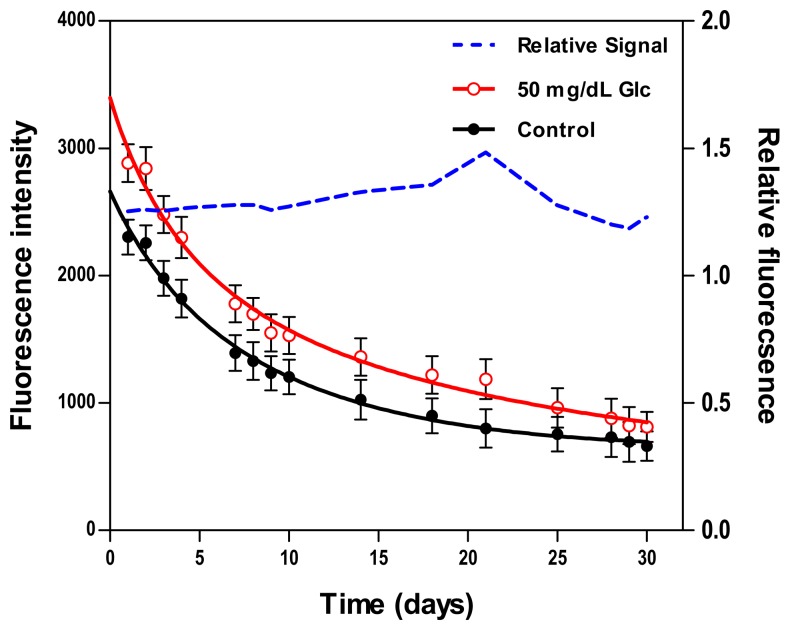
Demonstration of stability as exhibited by the absolute fluorescence response of the biosensor between 0 (solid circles, left *y*-axis) and 50 mg/dL (open circles, left *y*-axis) over 30 days. The dashed line (right *y*-axis) represents the relative daily glucose response normalized to intensity from corresponding control samples.

Two fluorescence measurements from biosensors resting in washing buffer and 50 mg/dL GC were periodically scanned over the course of 30 days. Figure 5 presents results from the daily testing, including absolute fluorescence intensity and relative fluorescence change due to glucose normalized to measurements from control samples. The total decay in the absolute fluorescence intensity over 30 days was 71.3% for the control sample and 71.8% for the 50 mg/dL sample. This apparent degradation in signal may be due to protein aggregation or sensing constituents leaching out of the hydrogel. Despite this monotonic decrease in the absolute fluorescence intensity, the relative response maintained a good stability over the course of 10 days, with a mean deviation of less than 2%, whereas beyond the 10-day period, this deviation was ±15%. Similar findings were concluded in prior studies where the relative fluorescent response remained stable over 11–30 days [52,53,58].

#### 3.2.7. Precision

We evaluated the biosensor within-run precision at GCs of 50 mg/dL, 120 mg/dL, and 400 mg/dL. The basic statistics, expressed in concentration, are shown in Table 2. The values of CV were 1.2%, 3.2%, and 5.1% in the presence of 50, 80, and 120 mg/dL glucose levels, respectively, which represent the biosensor repeatability. As replicate CVs of <5% for entire glucose ranges are common in sensing technologies [55], the current results should be considered acceptable.

#### 3.2.8. Bias and Error Analysis

A regression analysis of biosensor measurements showed good correlation (*r* = 0.991) with the true concentration over the entire validation data (*n* = 29), and a MARD of 6.7% (*r*^2^ = 0.982; *p* < 0.0001). This value represents the total error including, random and systematic effects, as the reference is the true glucose [35]. While data in this graph appears to show higher variability above 200 mg/dL than below, the MARD for measurements below and above this level was found to be nearly equivalent (6.5% *versus* 7.0%, respectively). This result, along with data in Table 2, which shows a small (<4%) change in CV with GC, may be an indication of a minor degradation in biosensor prediction accuracy with GC. Overall, the MARD results appear to be below a level needed for high clinical accuracy. A prior mathematical model which showed that there is a strong correlation between MARD and false hypoglycemic results also indicated that a MARD of <7.5% is needed to achieve a false positive rates of <10% and false negative rates of <5% [62].

**Figure 6.**
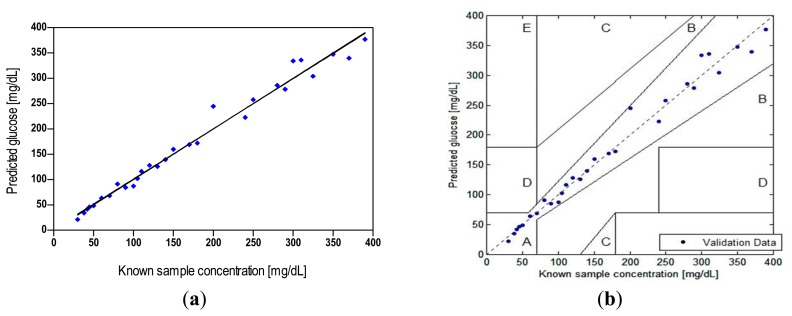
Accuracy of the biosensor with respect to known GC (*n* = 29). (**a**) Regression analysis, where line represents the perfect correlation; (**b**) Clarke's EGA.

Our construction of the error grid scatter plot is presented in Figure 6b, demonstrating the potential clinical significance of discrepancies between the biosensor results and true concentrations. The convention for various regions of the grid is as follows [45]. Zone “A” incorporates glucose readings that deviate from the reference by no more than 20% and would lead to clinically correct treatment decisions. Upper and lower zone “B” represents values that deviate from the reference by more than 20% but would lead to benign errors or no error in detection and treatment. The values falling in zone “C” would result in overcorrecting acceptable blood glucose levels, whereas zone “D” represents a significant failure to detect errors and would lead to clinically opposite treatment decisions.

Our assessment of the test against known samples (up to 400 mg/dL) showed that 100% of the biosensor readings (*n* = 29) fell in the innermost zone “A”, which compared favorably with the criterion that 95%, 5%, and 0% of the data should fall in zone “A”, “B”, and “C” or higher, respectively [49]. The result indicates that the deviation-related bias in our biosensor measurements can be characterized as clinically accurate—unlikely to lead to wrong treatment decisions; although a larger data set should yield a result that is more representative of the population and more statistically powerful. Furthermore, clinical implementation of FRET biosensors would likely introduce additional variability that would increase the level of error.

**Figure 7.**
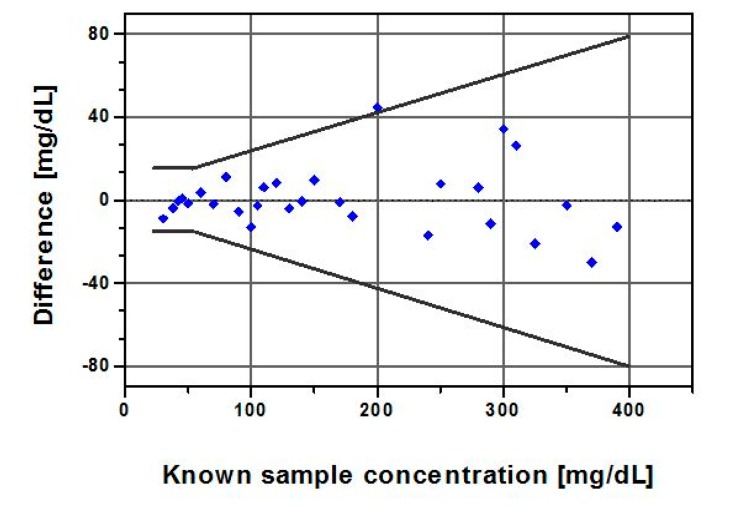
Accuracy plot demonstrates the difference between each individual glucose result falls within the acceptance limit. The two bold lines represent the ISO-15197 acceptance criteria.

The accuracy plot (Figure 7), which illustrates glucose difference between biosensor results and known samples against known glucose, showed that all individual glucose readings fell within the limits of the acceptance criteria described in the ISO 15197:2003. The standard differentiates between glucose levels below and above 75 mg/dL, as different criteria apply. It requires that 95% of the results fall within ±15 mg/dL or ±20% of the reference value for samples with GCs <75 mg/dL or ≥ 75 mg/dL, respectively.

The quantitative results (Tables 3 and 4) from the accuracy evaluation showed that the biosensor data classified per the ISO measures compared favorably with its 95% limits of accuracy acceptance criteria, although its measurement procedure differs. A total of 100% of our data fell within 9 mg/dL of true values for GCs < 75 mg/dL and 97% of the data for GCs ≥ 75 mg/dL fell within ±20%.

**Figure 8.**
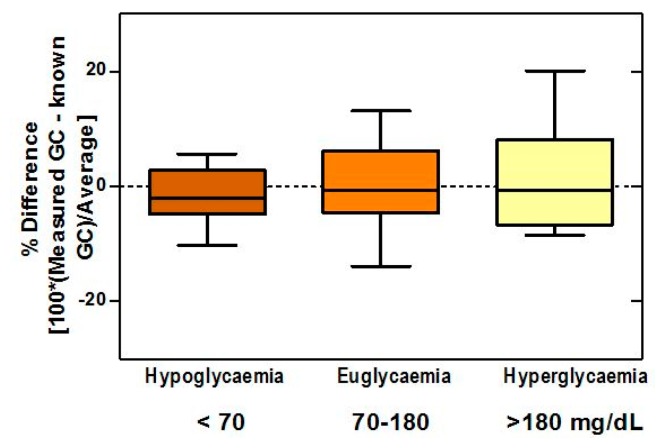
Boxplots of difference between the biosensor and true values demonstrate no outliers throughout the entire range. The bias percentages in hypo-, normo-, and hyperglycemic ranges were −1.62, −0.01, and 1.2, respectively.

The boxplots (Figure 8) of difference between the biosensor and true values showed bias percentages of −1.62, −0.01, and 1.2 in hypoglycemic, normoglycemic, and hyperglycemic ranges, respectively, and revealed no outliers throughout the entire range. The 95% limits of agreement for the respective ranges were −12.08 to 8.84, −14.63 to 14.60, and −16.09 to 18.58.

#### 3.2.9. Comparative Accuracy

**Figure 9.**
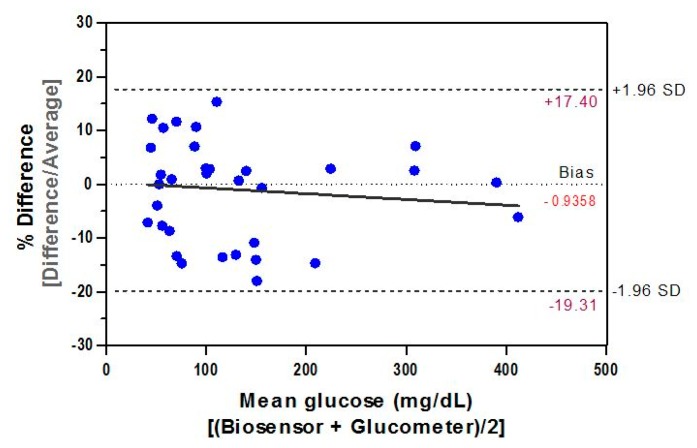
Bland-Altman difference plot (*n* = 34), showing the correlation between the biosensor and a commercial glucometer. The difference is plotted against mean values, and the 95% limits of agreement (dashed lines) of the difference between the two methods of measurement are shown, as is the regression line.

The difference plot of Bland-Altman was used to determine the mean difference between results from the biosensor and those obtained by the comparative method. The paired data set (*n* = 34) of our inter-comparison study represents a measurements' average by both methods and the result (Figure 9) demonstrated a significant (*r* = 0.993, *p* < 0.0001) correlation between them. The relative bias, biosensor minus glucometer, is −0.7444 mg/dL and the SD of bias is 9.95 mg/dL. Hence, the lower 95% agreement limit as compared to the comparative method is −20.25 mg/dL and the upper limit is 18.76 mg/dL. This indicates that, for 95% of data, the biosensor measurements will be between 20.25 mg/dL below the glucometer's measurement and 18.76 mg/dL above it. Such agreement estimation between measured data demonstrates equivalence between both methods and is within the ISO 95% performance criterion of the acceptance total error.

## 4. Conclusions

Based on a review of scientific literature and consensus documents for non-optical glucose technologies, a battery of test methods for the performance evaluation of FRET glucose biosensors was developed. Test characteristics included spectral response, linearity, sensitivity, limit of detection, kinetic response, reversibility, stability, precision, and accuracy—including an error grid analysis. Meanwhile, a protocol was developed and implemented for the fabrication of novel long-visible-wavelength FRET-based, glucose biosensors, with design parameters determined by a trade-off analysis. The test methods were implemented to fully characterize biosensor response *in vitro*. The effectiveness of the FRET biosensor was confirmed, with energy transfer efficiency of 0.98 and a response change of 45% in the presence of 400 mg/dL glucose. The biosensor demonstrated MARD of less than 11%, limit of detection of 25 mg/dL, and an average response time of 15 min. The loss of fluorescence signal—up to 72% over 30 days, however, was shown to be a significant concern.

The tests developed in this study provided a wide range of practical information on performance of our FRET glucose biosensors and have the potential to form the basis of rigorous standardized protocols for bench-top assessment of innovative optical technologies. In the future we intend to extend our research on this emerging technology to address test methods for assessing the potential impact of chemical interference and to assess the potential for optical interference by tissue chromophores and scatterers.

## Figures and Tables

**Table 1.** Summary of response characteristics and test methods used.

**Response Characteristic**	**Response Test Methods and Metrics**	**Reference**
Spectral Response	Plot of fluorescence spectra *vs.* glucose level	[19,20,39]
Calibration Quality Linearity Sensitivity	Plot of band-limited fluorescence *vs.* glucose level, correlation coefficient (*r*), slope, intercept Coefficient of determination (*r*^2^) Slope of calibration curve (%/mg/dL)	[19,40–43]
Limit of Detection	Absolute and relative SD and confidence level	[40]
Kinetic Response	Plot of predicted glucose over time in response to step increasing and decreasing of glucose level Response time (90% Response Time and 90% Fall Time)	[30,35]
Reversibility	Graphical demonstration of response over time, with stepwise increasing then decreasing glucose Hysteresis error	[30]
Stability	Evaluation of short-term variations Long-term fluorescence response/decay	[25,30]
Precision	Mean, SD, %CV (Coefficient of variation)	[36,43,44]
Bias	Plot of correlation and regression analysis of agreement with true GC_S_:Slope, intercept, *r*^2^, *r*, *p* MARD, 95% CI % Difference using accuracy plot % Difference using Boxplot	[36,44–47]
Error Analysis	Evaluation of clinical significance of bias using Clarke's error grid analysis (EGA)	[35,48–50]
Comparative Accuracy	% Difference using Bland-Altman plot (Correlation of difference; *r*, *p*)	[45,51]

**Table 2.** Results from repeatability evaluation at low, medium, and high GCs (*n* = 20).

	**Relative Fluorescence Intensity**		

	**GC = 50 mg/dL**	**GC = 120 mg/dL**	**GC = 400 mg/dL**
Mean	1.070	1.165	1.475
SD	0.0124	0.0369	0.0746
% CV	1.2	3.2	5.1

**Table 1.** Biosensor data classified per ISO 15197 and difference against true glucose.

**<75 mg/dL**		**≥75 mg/dL**	
	
Relative difference	No. of samples	Relative difference	No. of samples
≤ ±15 mg/dL	12/12 (100%)	≤ ±20%	35/36 (97%)

**Table 2.** Biosensor accuracy and performance.

**Glucose (mg/dL)**	**No. of Samples**	**Clarke A Zone**	**MARD**
≤50	8	8/8 (100%)	
51–80	4	4/4 (100%)	
81–120	8	8/8 (100%)	
121–240	8	8/8 (100%)	
≥241	20	20/20 (100%)	

All results	48	48/48 (100%)	10.42%
